# The Group B Streptococcal Adhesin BspC Interacts with Host Cytokeratin 19 To Promote Colonization of the Female Reproductive Tract

**DOI:** 10.1128/mbio.01781-22

**Published:** 2022-09-07

**Authors:** Haider S. Manzer, Dustin T. Nguyen, Joo Youn Park, Nogi Park, Keun Seok Seo, Justin A. Thornton, Angela H. Nobbs, Kelly S. Doran

**Affiliations:** a University of Colorado Anschutz Medical Campus, Department of Immunology and Microbiology, Aurora, Colorado, USA; b Mississippi State University, Department of Comparative Biomedical Sciences, College of Veterinary Medicine, Mississippi State, Mississippi, USA; c Mississippi State University, Department of Biological Sciences, Mississippi State, Mississippi, USA; d University of Bristol, Bristol Dental School, Bristol, United Kingdom; University of Illinois at Chicago

**Keywords:** anti-virulence, antigen I/II, cytokeratin-19, group B streptococcus, therapeutics, vaginal colonization

## Abstract

Streptococcus agalactiae, otherwise known as Group B Streptococcus (GBS), is an opportunistic pathogen that vaginally colonizes approximately one third of healthy women. During pregnancy, this can lead to *in utero* infection, resulting in premature rupture of membranes, chorioamnionitis, and stillbirths. Furthermore, GBS causes serious infection in newborns, including sepsis, pneumonia, and meningitis. Previous studies have indicated that GBS antigen (Ag) I/II family proteins promote interaction with vaginal epithelial cells; thus, we hypothesized that the Ag I/II Group B streptococcal surface protein C (BspC) contributes to GBS colonization of the female reproductive tract (FRT). Here, we show that a Δ*bspC* mutant has decreased bacterial adherence to vaginal, ecto-, and endocervical cells, as well as decreased auto-aggregation and biofilm-like formation on cell monolayers. Using a murine model of vaginal colonization, we observed that the Δ*bspC* mutant strain exhibited a significant fitness defect compared to wild-type (WT) GBS and was less able to ascend to the cervix and uterus *in vivo,* resulting in reduced neutrophil chemokine signaling. Furthermore, we determined that BspC interacts directly with the host intermediate filament protein cytokeratin 19 (K19). Surface localization of K19 was increased during GBS infection, and interaction was mediated by the BspC variable (V) domain. Finally, mice treated with a drug that targets the BspC V-domain exhibited reduced bacterial loads in the vaginal lumen and reproductive tissues. These results demonstrate the importance of BspC in promoting GBS colonization of the FRT and that it may be targeted therapeutically to reduce GBS vaginal persistence and ascending infection.

## INTRODUCTION

Streptococcus agalactiae, otherwise known as Group B Streptococcus (GBS), colonizes approximately one third of healthy women and is vertically transmitted to up to 70% of infants born to GBS-positive mothers (1, 2). Approximately 1 to 2% of newborns will develop invasive disease, making GBS a leading cause of neonatal sepsis, pneumonia, and bacterial meningitis worldwide (2, 3). Furthermore, GBS colonization itself is a risk factor for many adverse pregnancy outcomes. GBS contributes to 1% of all stillbirths, or roughly 26,000 each year in developed nations and four times that amount throughout Africa (4). Preterm premature rupture of membranes (PPROM) has also been shown to be 3.6 times more likely in pregnant women that are GBS positive (5). In addition, GBS is highly associated with chorioamnionitis, or the inflammation of intrauterine structures ([Bibr B6][Bibr B7][Bibr B8]). Despite these numerous and serious complications associated with GBS colonization during pregnancy, the mechanisms that allow GBS to colonize the vaginal tract or cause intrauterine infection are not well understood. Only a few GBS surface proteins have been shown to contribute to vaginal cell adherence and GBS colonization *in vivo*. These include factors that are known to interact with extracellular matrix components (ECM) such as serine-rich repeat proteins (Srr-1, Srr-2), pili, and the plasminogen binding surface protein (PbsP) ([Bibr B9][Bibr B10][Bibr B12]). A greater understanding of the molecular interactions between GBS adhesins and specific host receptors would facilitate development of anticolonization therapeutics.

The Group B streptococcal surface proteins (Bsp) are members of the Antigen type I/II (AgI/II) family of multifunctional adhesins. Strains within the majority of Streptococcus species possess genes encoding AgI/II proteins, which have been implicated in promotion of adherence to various surfaces, including teeth, lung epithelium, and the blood-brain barrier (BBB) endothelium (13, 14). The GBS Bsp genes have 4 known homologs, BspA–BspD, which have been identified in ~27% of GBS genomes, with 55% of these being BspC (15). We recently showed that BspC adherence to the BBB contributes to the pathogenesis of GBS meningitis (16). BspC interacts directly with the type-III intermediate filament protein known as vimentin, which is highly expressed in BBB endothelial cells (17, 18). The BspC-vimentin interaction is mediated by a ligand-binding pocket guarded by a gating loop contained in the BspC variable (V-) domain (15). Bsp proteins have also been shown to be necessary for optimal GBS adherence to vaginal epithelial cells (19). Furthermore, BspA and BspC proteins are sufficient to promote adherence to vaginal epithelial cells when each is individually heterologously expressed in Lactococcus lactis (19, 20). We sought to further characterize the role of BspC during *in vivo* colonization and to define the molecular interactions involved.

Here, we demonstrate that a Δ*bspC* mutant exhibits reduced aggregation and is defective in adherence to vaginal and cervical cells. We also show for the first time that BspC contributes to GBS vaginal colonization within an *in vivo* murine model. The Δ*bspC* strain exhibited decreased ascension to the cervix and uterus, as well as decreased inflammatory signaling in the female reproductive tract (FRT) compared to the WT strain. Furthermore, we show that GBS infection induces increased surface localization of cytokeratin 19 (K19) and identify K19 as a main BspC receptor. This interaction is mediated by the ligand-binding pocket of the V-domain. Finally, our results demonstrate the BspC can be targeted therapeutically to limit GBS in the vaginal tract and decrease ascending infection.

## RESULTS

### BspC contributes to GBS adherence and aggregation.

**FIG 1:**
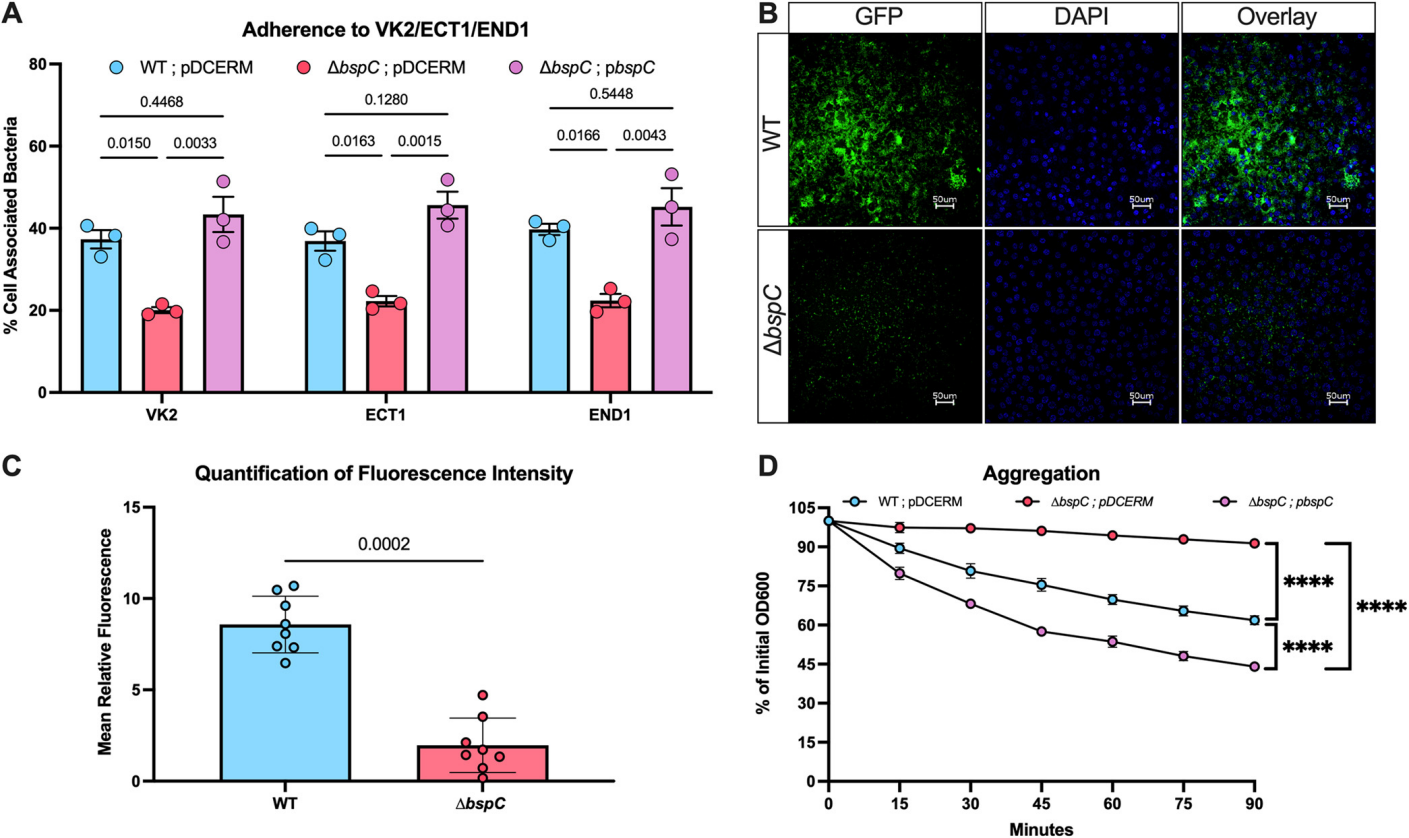
BspC contributes to GBS adherence and aggregation. (A) Adherence of COH1 pDCErm, COH1 Δ*bspC* pDCErm, and COH1 Δ*bspC* pDCErm::*bspC* to vaginal (VK2), ectocervical (ECT1), and endocervical (END1) cells was assessed 30 min after incubation. (B) VK2 cells were infected with GFP expressing WT and Δ*bspC* for 24 h prior to nuclear staining with DAPI and imaging. Two to three images were taken per condition per experiment, and representative images from one of three independent experiments are shown. (C) Fluorescence intensity from the full set of microscopy images was quantified using ImageJ. (D) Aggregation of GBS was assessed based on supernatant measured at OD600 from standing cultures over time. A, C, and D display pooled data from three independent experiments, and error bars represent the SEM. Statistical analysis: one-way ANOVA with Sidak’s multiple comparisons (A), unpaired *t* test (C), and two-way ANOVA with Tukey’s multiple comparisons (D). ****, *P* < 0.00005.

It was previously shown that GBS utilizes Bsp proteins to adhere to vaginal epithelial cells (19, 20). As serotype III ST-17 strains such as COH1 are highly associated with invasive disease (21), we sought to confirm this phenotype with the hypervirulent strain COH1 that expresses BspC, as well as assess the contribution to GBS interaction with human cervical epithelial cells. To this end, we measured adherence of COH1 WT, Δ*bspC*, and a complemented strain (p*bspC*) to human vaginal epithelial cells (VK2), human ectocervical cells (ECT1), and human endocervical cells (END1). We observed a significant reduction in the ability of the Δ*bspC* mutant strain to adhere to all three cell lines relative to both WT and complemented strains (Fig. 1A).

We previously observed using SEM that WT GBS had increased bacterial cell–cell interactions compared to the Δ*bspC* mutant (16), and as these phenotypes have the potential to increase persistence of GBS within the vaginal tract, we used fluorescence microscopy to visualize GBS after 24 h of growth on the surface of vaginal epithelial cells (Fig. 1B). WT GBS formed a mat of bacteria that persisted on the vaginal epithelium despite vigorous washing. Quantification of GBS fluorescence confirmed that the Δ*bspC* mutant was quite sparse compared to WT (Fig. 1C). Based on the thick mat of WT bacterial growth, we next aimed to quantify the role of BspC in GBS auto-aggregation as described in Materials and Methods. We assessed the OD600 of standing GBS cultures over time and observed that the Δ*bspC* strain remained in suspension relative to the WT and complemented strains, suggesting a decreased ability to form aggregates (Fig. 1D). The complemented strain exhibited increased aggregation compared to WT GBS, likely due to overexpression of BspC with a constitutive promoter in this plasmid-expression system. Collectively, these *in-vitro* phenotypes led us to hypothesize that BspC may contribute to GBS colonization and persistence within the FRT *in vivo*.

### BspC promotes colonization and ascending infection.

**FIG 2:**
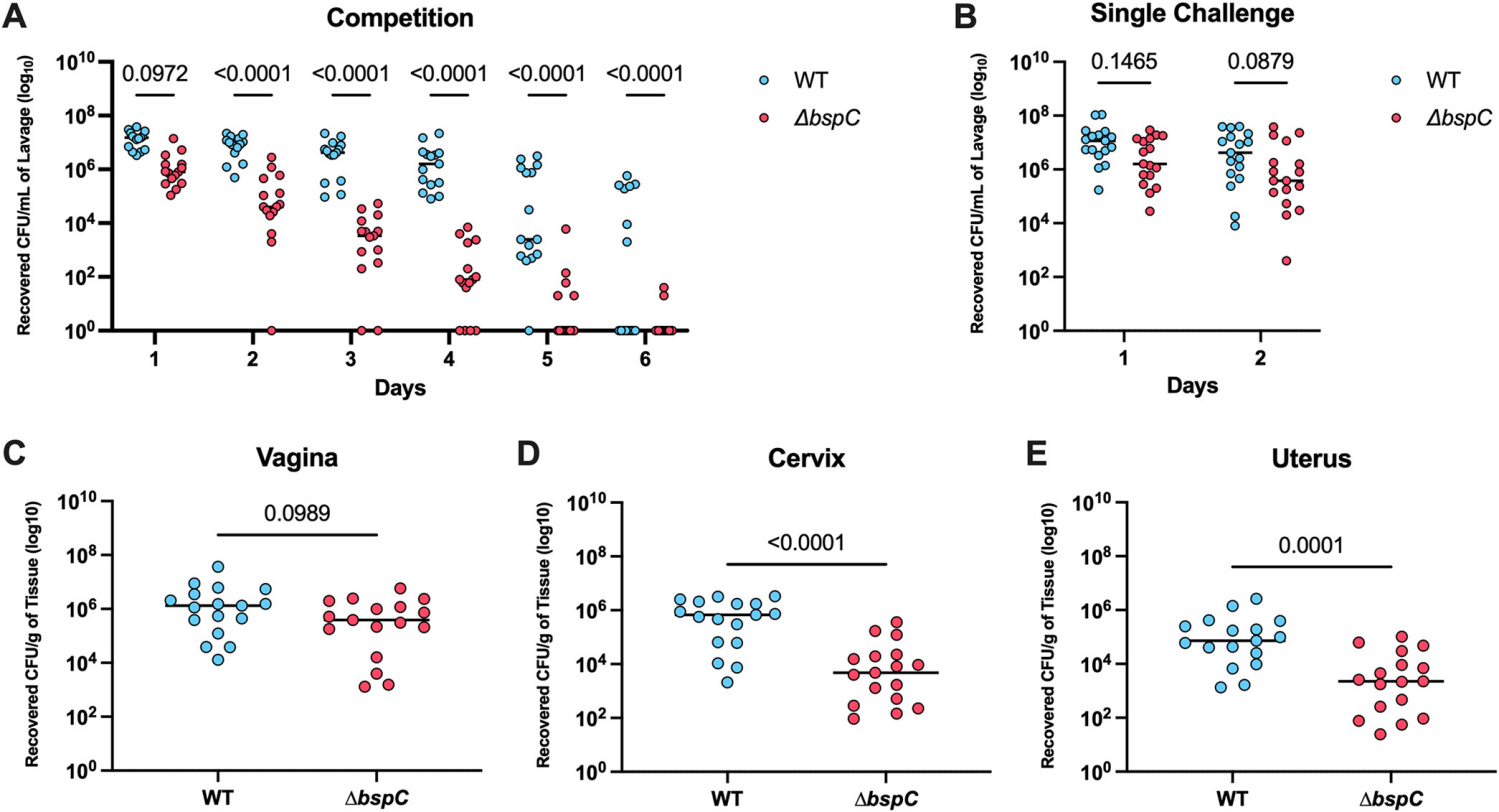
BspC contributes to GBS colonization and ascending infection. (A) Mice were coinoculated directly into the vaginal tract with 10^7^ CFU of WT and Δ*bspC* GBS. Recovered CFU counts from daily swabs are shown. Data are pooled from two independent experiments, *n* = 5 to 10 mice per group in each experiment. (B to E) Separate groups of mice were used for direct inoculation of 2 × 10^7^ CFU of either WT or Δ*bspC* GBS into the murine vagina. (B) Recovered CFU counts from daily swabs are shown. Forty-eight hours postinoculation, the vagina (C), cervix (D), and uterus (E) tissues were harvested, homogenized, and plated to enumerate CFU. Solid horizontal lines indicate the median, and each dot represents an individual mouse. Data from B to E are pooled from two independent experiments, *n* = 7 to 10 mice per group in each experiment. Statistical analysis: two-way ANOVA with Tukey’s multiple comparisons (A and B) and unpaired *t* test (C to E).

To investigate the role of BspC in GBS FRT colonization *in vivo*, we used our murine model of GBS colonization ([Bibr B22][Bibr B23][Bibr B25]). CD-1 mice were vaginally inoculated with both WT and the Δ*bspC* mutant in competition, and then swabbed daily to quantify recovered bacterial CFU. The Δ*bspC* mutant strain exhibited a significant fitness defect compared to WT GBS as early as day 2 post-colonization. Furthermore, the Δ*bspC* strain was outcompeted and cleared from the vaginal tract faster than the WT strain (Fig. 2A). By day 5, only 33% of mice retained the Δ*bspC* mutant compared to 93% of mice that were still colonized with WT GBS (Fig. S1A in the supplemental material). We observed similar results using a different mouse background and a Δ*bspC* mutant generated in a different GBS strain background (Fig. S1B and C). As BspC contributed to GBS interaction with other FRT cell types, we hypothesized that BspC may also impact the ability of GBS to colonize and ascend to higher tissues *in vivo*. To test this, groups of mice were inoculated with either WT or the Δ*bspC* mutant, and the vaginal, cervical, and uterine tissues were harvested 48 h post-inoculation. At this time point there were no significant differences in recovered CFU between the WT and Δ*bspC* in the vaginal lumen or vaginal tissue (Fig. 2B, C); however, we observed significantly reduced bacterial loads in the cervix and uterus in Δ*bspC* colonized mice (Fig. 2D, E). Together, these data indicate that BspC is important for GBS fitness in the vaginal tract as well as ascending spread to the cervix and uterus.

10.1128/mbio.01781-22.1 FIG S1 BspC contributes to colonization. (A) WT or Δ*bspC* GBS recovered from the vaginal lumen within each mouse from the competition experiment shown in Fig. 2A is shown here as a percent per mouse. (B)10^7^ CFU each of GBS WT and Δ*bspC* in the COH1 background were coinoculated directly into the murine vaginal tract of 129S mice. Recovered CFU counts from daily swabs are shown. (C) 10^7^ CFU each of GBS WT and Δ*bspC* in the 515 background were coinoculated directly into the murine vaginal tract of CD-1 mice. Recovered CFU counts from daily swabs are shown. (D) Adherence of WT, Δ*bspC*, and rifampicin-resistant Δ*bspC* GBS to vaginal (VK2) cells was assessed 30 minutes after incubation. (E) WT, Δ*bspC*, and rifampicin-resistant Δ*bspC* GBS were grown for 8 h in THB, and the OD600 was measured every hour. Statistical analysis: two-way ANOVA with Tukey’s multiple comparisons (B, C, and E); one-way ANOVA with Sidak’s multiple comparisons (D). **, *P* < 0.005; ***, *P* < 0.0005. Download FIG S1, TIF file, 0.5 MB. Copyright © 2022 Manzer et al. 2022 Manzer et al. https://creativecommons.org/licenses/by/4.0/ This content is distributed under the terms of the Creative Commons Attribution 4.0 International license.

### Contribution of BspC to neutrophil signaling.

**FIG 3:**
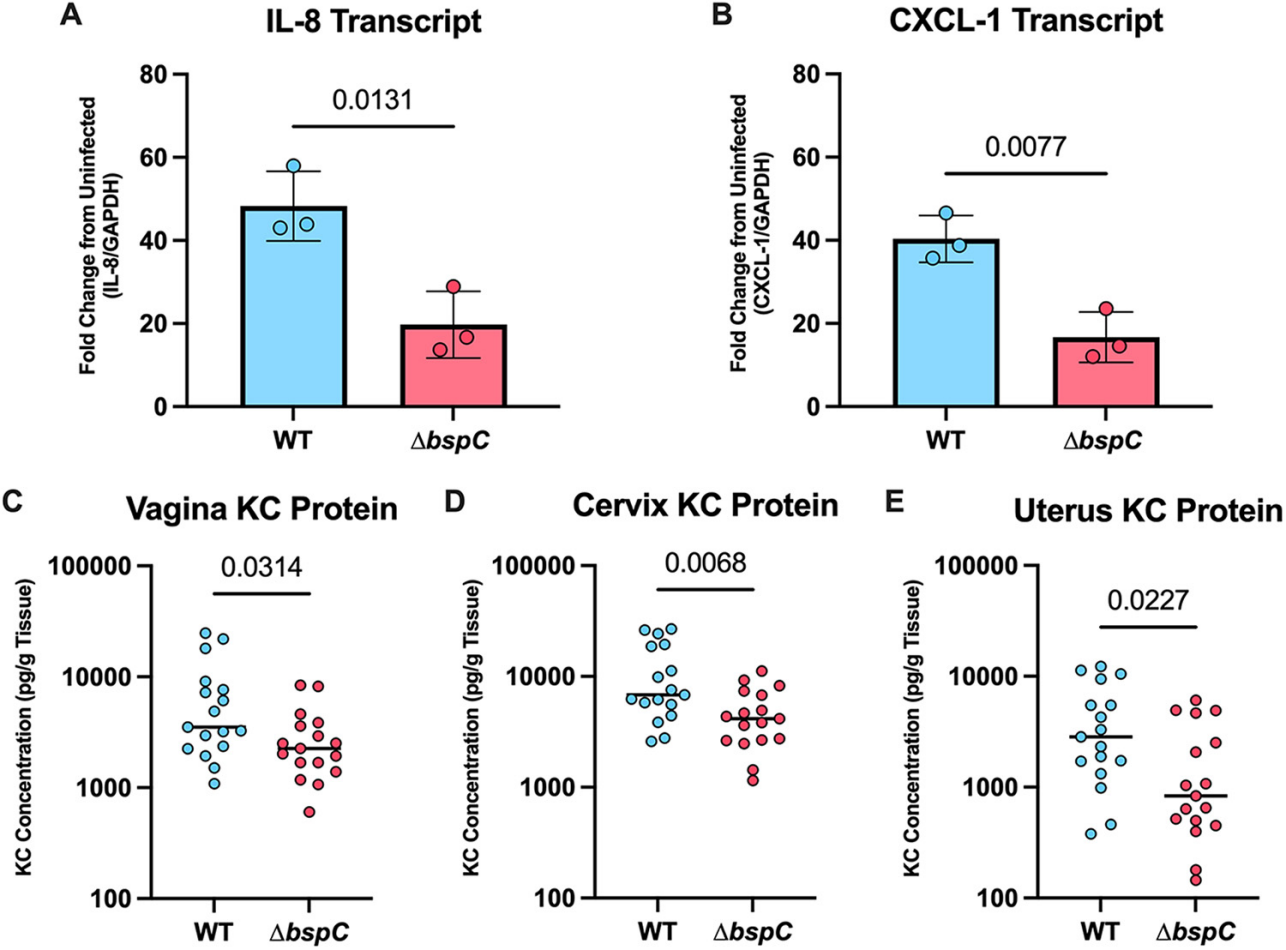
BspC contributes to inflammation in the FRT. Vaginal (VK2) cells were infected with 1 × 10^6^ CFU (multiplicity of infection [MOI] of 10) of either WT or Δ*bspC* GBS for 4 h prior to isolation of RNA. Fold changes of IL-8 (A) and CXCL-1 (B) from infected cells are shown relative to uninfected cells and normalized to GAPDH expression. (C to E) WT and Δ*bspC* GBS were inoculated directly into the murine vaginal tract in single challenge. KC protein in the vaginal tract (C), cervix (D), and uterus (E) were measured via ELISA 48 h postinoculation. In A and B, data are pooled from three independent experiments where each dot represents the mean from three technical replicates, the bar indicates the mean across the independent experiments, and error bars represent the SEM. In C to E, pooled data from two independent experiments, *n* = 7 to 10 mice per group in each experiment, are shown where horizontal lines indicate the median, and each dot represents an individual mouse. Statistical analysis: unpaired *t* tests (A to E).

We have previously shown that BspC is necessary and sufficient to induce neutrophil chemokine signaling in brain endothelial cells and during the progression of meningitis *in vivo* (15, 16). As neutrophil influx is associated with chorioamnionitis and preterm labor after bacterial ascending infection (8, [Bibr B26][Bibr B27][Bibr B28]), we sought to investigate the proinflammatory potential of BspC within this niche. We infected human vaginal epithelial cells with GBS WT or the Δ*bspC* mutant for 4 h, then harvested RNA for analysis of neutrophil chemokine transcript abundance. WT GBS infection led to a significant increase in IL-8 and CXCL-1 compared to the Δ*bspC* mutant and uninfected controls (Fig. 3A and B). To confirm these results, we also analyzed protein abundance of KC (the murine equivalent of CXCL-1) in vaginal, cervical, and uterine tissues of mice 48 h after GBS infection. We observed significantly more KC in all tissues during infection with WT GBS compared to tissues recovered from mice infected with the Δ*bspC* mutant strain (Fig. 3C to E).

### Identification of cytokeratin-19 as a BspC host receptor.

**FIG 4:**
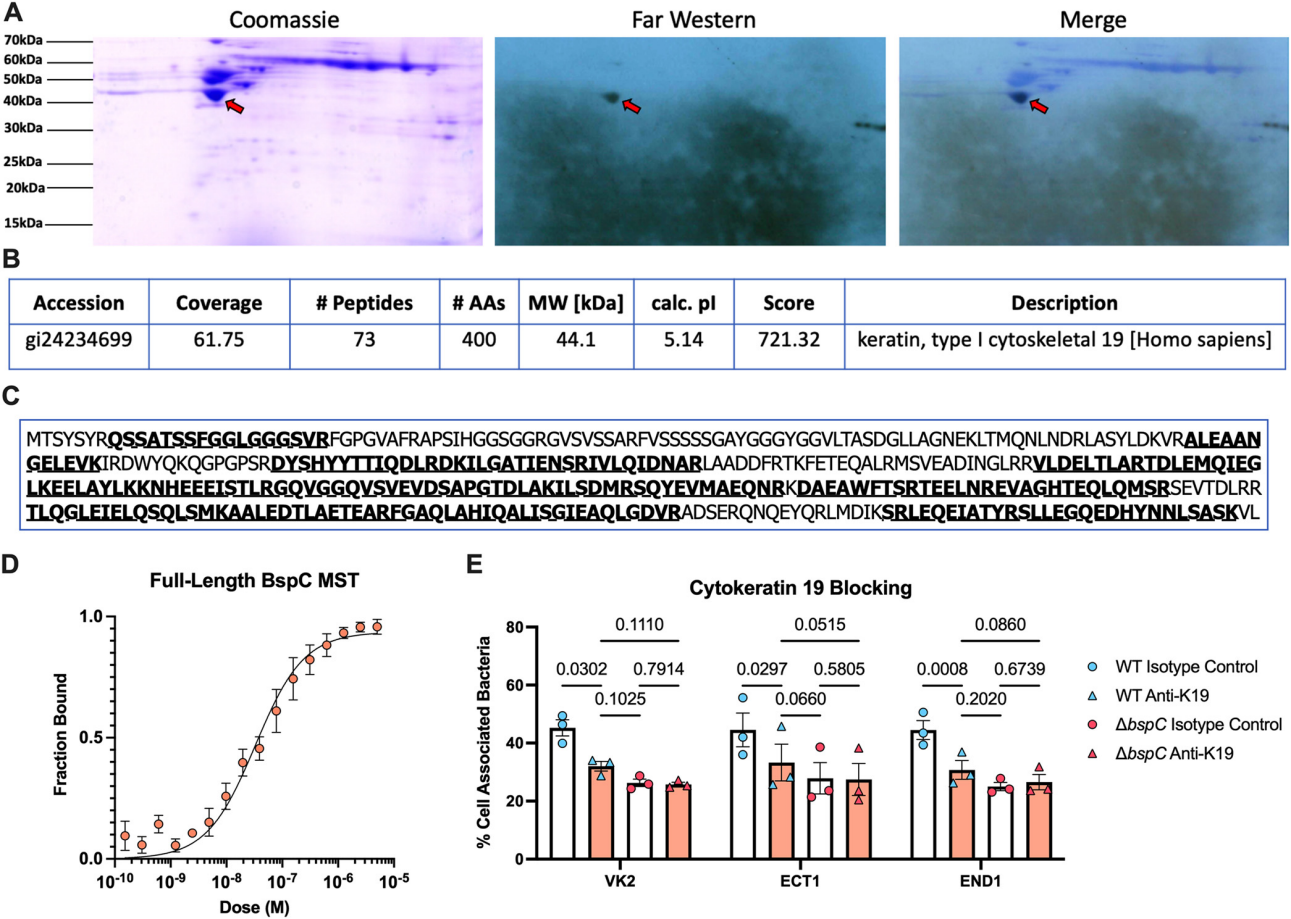
BspC interacts with cytokeratin 19. (A) Membrane proteins were extracted from vaginal epithelial (VK2) cells and separated by 2-D electrophoresis in duplicate. One gel was stained with Coomassie blue, while the other was transferred to a PVDF membrane and probed with biotinylated BspC. The specific interaction of BspC was detected by a streptavidin antibody conjugated to HRP and visualized by X-ray film exposure. The spot highlighted by the red arrow was identified from the X-ray film that was aligned to the Coomassie stained gel. The spot shown with the red arrow in A was excised and digested with trypsin for electrospray ionization-tandem mass spectrometry analysis, which identified Cytokeratin 19. (C) The protein sequence for Cytokeratin 19 is shown. The specific peptides that were identified via mass spectrometry are shown in bold and underlined. (D) Microscale thermophoresis was performed with His-tagged Cytokeratin 19 at 25 nM and the indicated concentrations of full-length BspC protein. A dose-response curve of the fraction of bound protein using pooled data from four replicates is shown. (E) Vaginal (VK2), ectocervical (ECT1), and endocervical (END1) cells were pretreated with either an anti-cytokeratin 19 antibody or the isotype control 30 min prior to infection with either wild-type or Δ*bspC* GBS. Adherence was assessed 30 min after infection. Pooled data from three independent replicates are shown. Each dot represents the mean of four technical replicates in each independent experiment; bars indicate the mean of all three experiments. All error bars indicate the SEM. Statistical analysis: two-way ANOVA with Tukey’s multiple comparisons (E).

We previously identified that the host receptor for BspC in BBB endothelial cells is the intermediate filament protein known as vimentin (16). Epithelial cells typically have low expression of vimentin; therefore, we hypothesized that BspC must interact with an alternative host receptor within this niche. To identify a receptor in vaginal epithelial cells, VK2 membrane proteins were separated by 2-dimensional electrophoresis and probed with biotinylated BspC protein. A far Western blot revealed one primary interaction between BspC and a ~44 kDa membrane protein (Fig. 4A). This spot was excised, and mass spectrometry was used to identify the protein to be cytokeratin-19 (K19), which is in the intermediate filament protein family (Fig. 4B, C). To confirm this interaction, we used MicroScale Thermophoresis (MST), which uses the altered thermal response of a bound protein to generate a dose-response curve and quantify strength of direct protein–protein interactions. The MST results confirmed that full-length BspC protein interacts directly with K19 with an estimated *K*_d_ of 32.9 nM (Fig. 4D). To investigate the contribution of the BspC-K19 interaction to GBS adherence to FRT cells, we pretreated vaginal, ectocervical, and endocervical cells with an anti-K19 antibody prior to infection with GBS. Pretreatment with the anti-K19 antibody reduced BspC-dependent adherence of GBS to all three cell lines compared to an isotype control antibody pretreatment. These results confirm that GBS utilizes the BspC-K19 interaction to facilitate adherence to cells within the FRT (Fig. 4E).

**FIG 5:**
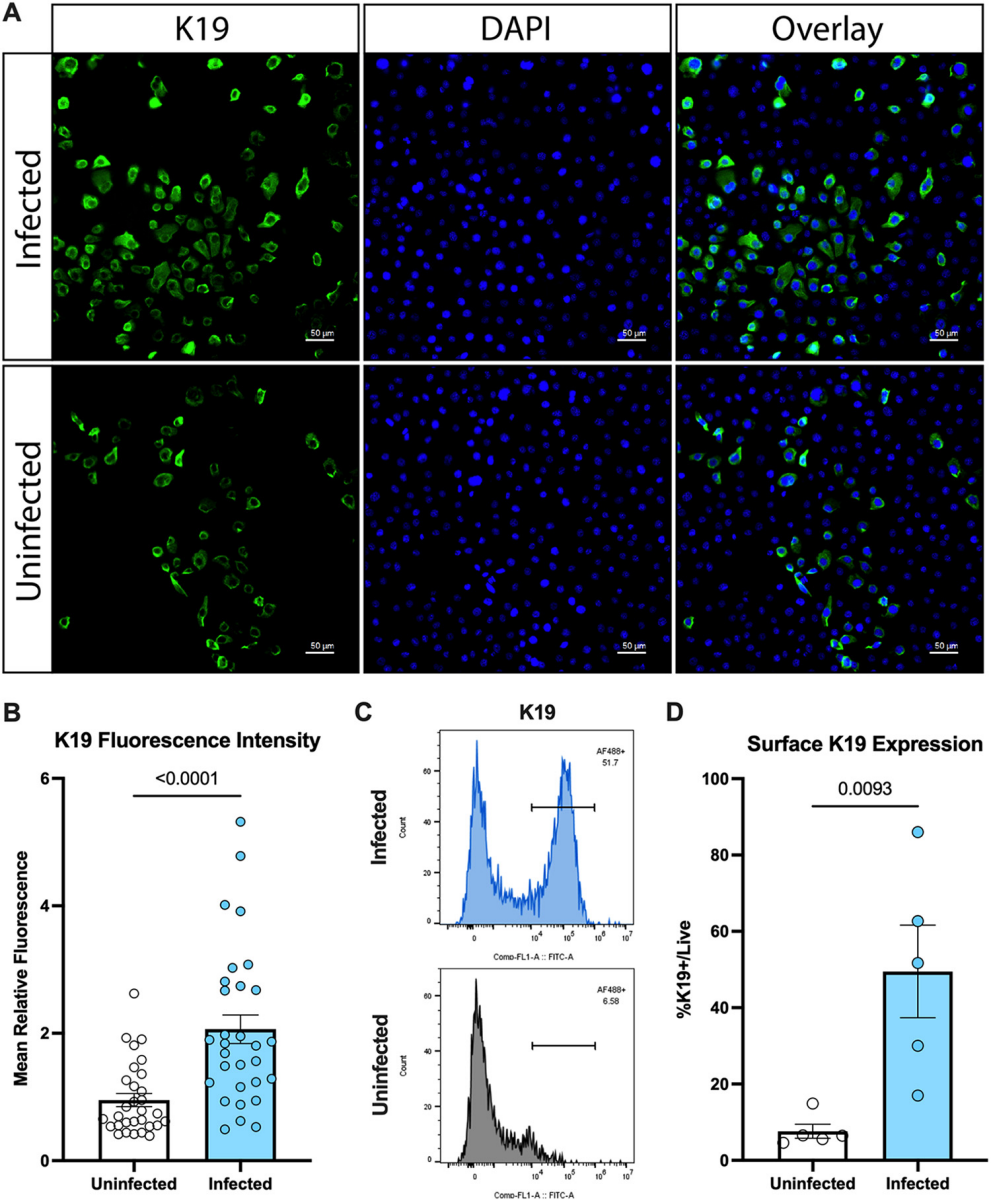
GBS infection induces VK2 K19 surface localization. (A) Vaginal (VK2) cells were either uninfected or infected with WT GBS. After 24 h of incubation, cells were washed 5× with PBS, fixed with PFA, and then stained for surface K19 (green) and DAPI (blue) prior to imaging. Ten images were taken per condition per experiment, and representative images from one of three independent experiments are shown. (B) The mean fluorescence intensity from the channel detecting K19 from all images taken in A was quantified using ImageJ. (C) Vaginal (VK2) cells were either uninfected or infected with WT GBS. After 24 h of incubation, cells were washed 5× with MACS buffer and then stained with a membrane-impermeable live/dead dye and the K19 antibody. Cells were then fixed with PFA, and flow cytometry was used to detect K19 staining (AF488) after gating on live cells. Representative histograms from one of five independent experiments are shown. (D) Quantification of the percentage of live cells that were positive for surface K19 (AF488) staining from C. In B and D, bars indicate the mean of all independent experiments, and error bars indicate the SEM. Statistical analysis: unpaired *t* tests (B and D).

Despite being traditionally thought of as cytosolic due to their structural roles, cytokeratin proteins have been known to localize to the cell surface ([Bibr B29][Bibr B30][Bibr B31]). To our knowledge, however, this has not been confirmed in vaginal epithelial cells. To investigate surface localization of K19, we stained uninfected and GBS-infected vaginal epithelial cells with an anticytokeratin 19 antibody, without permeabilization, and observed that a subset of cells were positive for K19. Interestingly, the number of surface K19+ cells seemed to increase upon infection with GBS (Fig. 5A, B). Staining with an isotype control antibody resulted in no fluorescence (Fig. S2A). We also investigated this using flow cytometry while including a membrane-impermeable live/dead dye and confirmed that the number of live surface-K19+ cells was increased upon infection (Fig. 5C, D). This increase was not solely due to an increase in total K19 protein levels, as Western blot analysis revealed only a modest increase in K19 protein after infection (Fig. S2B and C). These data indicate that GBS infection induces K19 surface localization on vaginal epithelial cells, allowing for interaction with BspC to promote adherence and colonization.

10.1128/mbio.01781-22.2 FIG S2 Microscopy control and K19 Western blot. (A) Vaginal (VK2) cells were either uninfected or infected with WT GBS. After 24 h of incubation, cells were washed 5× with PBS, fixed with PFA, and then stained with an IgG isotype control antibody (green) and DAPI (blue) prior to imaging. Representative images from one of three independent experiments are shown. (B) Vaginal (VK2) cells were either uninfected or infected with WT GBS. After 24 h of incubation, cells were washed 5× with MACS buffer and then stained with a membrane-impermeable live/dead dye and an IgG isotype control antibody. Cells were then fixed with PFA and flow cytometry was used to detect AF488 after gating on live cells. Representative histograms from one of five independent experiments are shown. (C) Vaginal (VK2) cells were either uninfected or infected with WT GBS. After 24 h of incubation, cells were washed 5× with PBS and then lysed. Cell lysates were run on an SDS-PAGE gel for western blotting of both whole-cell K19 and GAPDH. Blots from all three independent experiments are shown. (D) Blot intensity from all three experiments for C were quantified. Uninfected samples were normalized to 1, and the fold change of K19 protein levels in the infected cells relative to GAPDH over uninfected cells is shown. In D, bars indicate the mean of all three experiments, and error bars indicate the SEM. Statistical analysis: unpaired *t* test (D). Download FIG S2, TIF file, 0.6 MB. Copyright © 2022 Manzer et al. 2022 Manzer et al. https://creativecommons.org/licenses/by/4.0/ This content is distributed under the terms of the Creative Commons Attribution 4.0 International license.

### Importance of the BspC V-domain.

**FIG 6:**
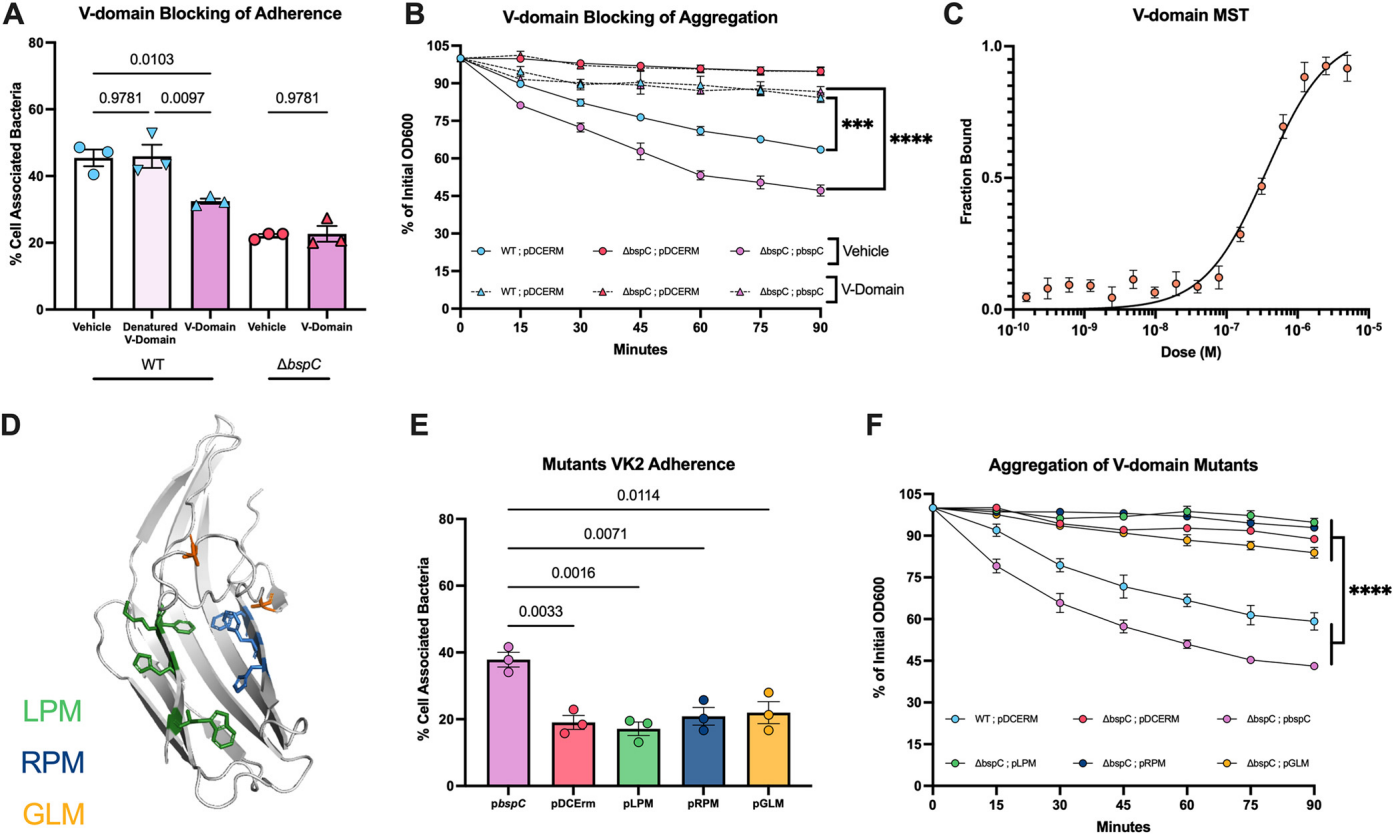
BspC-dependent adherence and aggregation are mediated by the V-domain. (A) Vaginal (VK2) cells were treated with 10-μM V-domain protein, heat-denatured V-domain protein, or the PBS vehicle 30 min prior to infection with WT or Δ*bspC*, and adherence was assessed after 30 min of incubation. (B) Either 0.1 mg/mL of V-domain protein or the PBS vehicle was added to GBS cultures, and aggregation of GBS was assessed based on diminishing supernatant OD600 as standing cultures settled to the bottom of the tube over time. (C) Microscale thermophoresis was performed with His-tagged Cytokeratin 19 at 25 nM and the indicated concentrations of the BspC V-domain. A dose-response curve of the fraction of bound protein is shown. (D) Diagram of the WT BspC V-domain visualized using PyMOL (59). The mutated residues are shown as sticks and color coded based on mutation, where green residues were mutated to generate LPM, blue residues were mutated to generate RPM, and orange residues were mutated to generate GLM. (E) Adherence of Δ*bspC* GBS complemented with either WT or V-domain mutant *bspC* strains to vaginal (VK2) cells was assessed 30 min after incubation. (F) Aggregation of GBS was assessed based on diminishing supernatant OD600 as standing cultures settled to the bottom of the tube over time. A, B, E, and F display pooled data from three independent experiments. In A and E, each dot represents the mean of four technical replicates in each independent experiment, bars indicate the mean of all three experiments, and error bars indicate the SEM. C displays pooled data from four independent experiments, and error bars indicate the SEM. Statistical analysis: one-way ANOVA with Sidak’s multiple comparisons (A and E); two-way ANOVA with Tukey’s multiple comparisons (B and F). ***, *P* < 0.0005; ****, *P* < 0.00005.

We recently determined that the BspC V-domain binding pocket was critical for BspC-dependent interaction with brain endothelial cells and vimentin (15). We hypothesized that the V-domain may similarly contribute to BspC-mediated adherence to FRT cells, auto-aggregation, and K19 interaction. We purified recombinant BspC protein consisting of only the V-domain. We then used the V-domain protein to pretreat VK2 monolayers prior to infection with GBS and observed BspC-dependent blocking of adherence to vaginal cells (Fig. 6A). Pretreatment with heat-denatured V-domain had no impact on WT GBS adherence. Previous studies have shown V-domain-dependent intermolecular interactions of the Streptococcus mutans AgI/II protein, which contributes to functional amyloid formation during biofilm growth ([Bibr B32][Bibr B33][Bibr B36]). As such, we hypothesized that the V-domain of BspC might also contribute to the auto-aggregation phenotype we observed earlier. To test this, we pretreated GBS cultures with the V-domain protein prior to measuring aggregation and observed that addition of V-domain protein prevented GBS aggregation in a BspC-dependent manner (Fig. 6B). We next used MST to determine whether the BspC V-domain alone could form the direct interaction with cytokeratin 19. We confirmed a direct BspC V-domain–K19 interaction with an estimated *K*_d_ of 362 nM (Fig. 6C), although this *K*_d_ was higher than the full-length BspC protein.

After confirming the importance of the V-domain in these interactions, we aimed to investigate a ligand-binding pocket contained within the V-domain. Previously, we used site-directed mutagenesis to create three multipoint mutants that alter key residues that form the ligand-binding pocket: left pocket mutant (LPM; F267A, F269A, and H271A), right pocket mutant (RPM; F379A, K380E, H382A, and W384A), and gating loop mutant (GLM; A250P/A259P) (15). The locations of the specific residues mutated in each of these strains are shown in Fig. 6D. A Δ*bspC* mutant strain complemented by the LPM, RPM, or GLM mutations on otherwise WT full-length BspC failed to restore adherence of GBS to vaginal epithelial cells to the same level as a strain complemented with WT BspC (Fig. 6E). We also measured the aggregation of these mutant strains and observed decreased aggregation compared to the WT or complement strains (Fig. 6F). These data indicate that the BspC-mediated aggregation and adherence to vaginal epithelial cells is dependent on the ligand-binding pocket contained within the BspC V-domain.

### Inhibition of BspC reduces GBS colonization.

**FIG 7:**
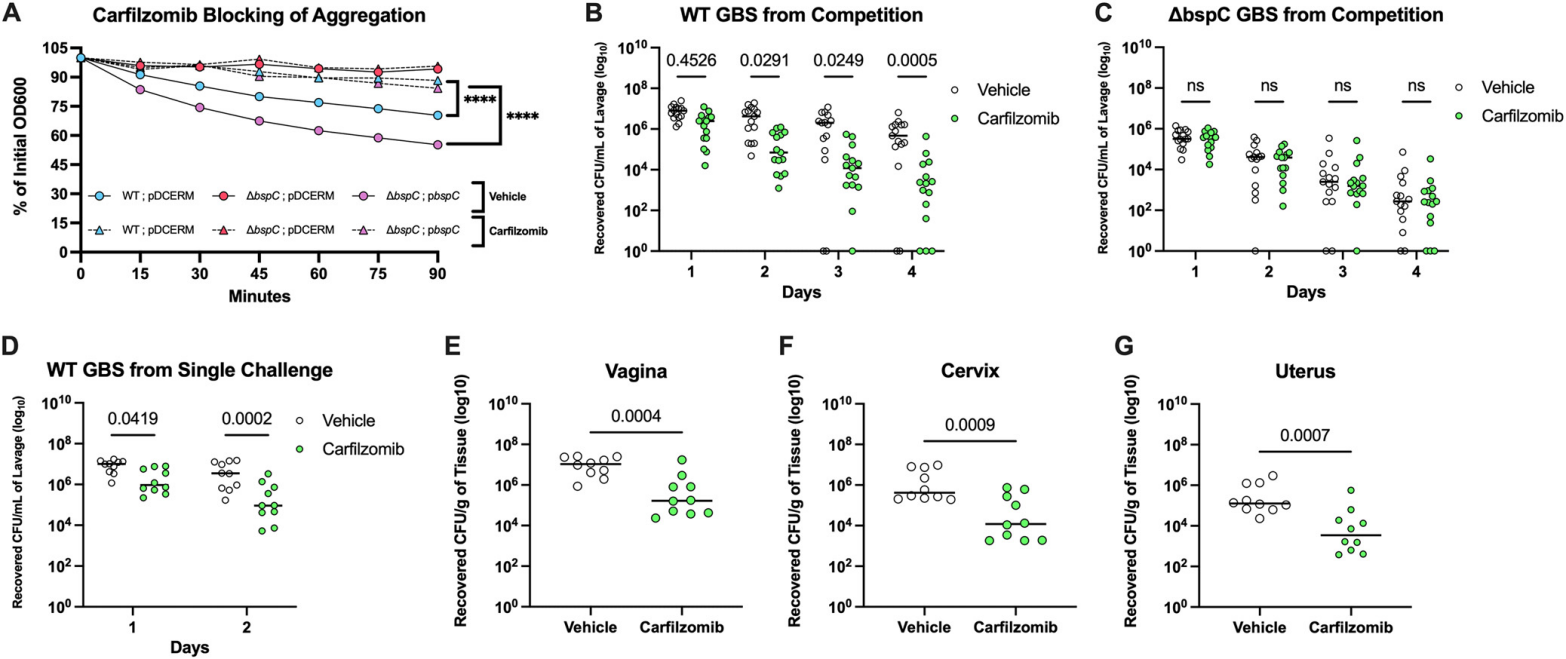
Carfilzomib treatment promotes reduction of GBS FRT colonization. (A) Either 1 μM carfilzomib or the DMSO vehicle was added to GBS cultures, and aggregation of GBS was assessed based on diminishing supernatant OD600 as standing cultures settled to the bottom of the tube over time. (B) 10^7^ CFU each of GBS WT and Δ*bspC* were coinoculated directly into the murine vaginal tract. Mice were treated with 35 μg of carfilzomib or the vehicle directly into the vaginal lumen each day. Recovered CFU counts of WT GBS from daily lavages are shown. Data are pooled from three independent experiments, *n* = 5 mice per group in each experiment. (C to G) 2 × 10^7^ CFU of WT GBS was inoculated directly into the murine vaginal tract in single challenge, and then mice were treated with 35 μg of carfilzomib or the vehicle directly into the vaginal lumen each day. Recovered CFU counts from daily lavages are shown (D). GBS CFU counts from the vagina (E), cervix (F), and uterus (G) 48 h postinoculation are shown. Data from B to E are pooled from 2 independent experiments, *n* = 5 mice per group in each experiment. Solid horizontal lines indicate the median, and each dot represents an individual mouse. Statistical analysis: two-way ANOVA with Tukey’s multiple comparisons (A to C) and unpaired *t* test (D to G). ****, *P* < 0.00005.

We previously showed that the BspC V-domain ligand-binding pocket was important for the pathogenesis of GBS meningitis, and then used a structure-based virtual drug screen to identify drugs that might bind the V-domain pocket (15). We showed that one drug from this screen, carfilzomib, was able to block BspC-dependent adherence of GBS to BBB endothelium and decrease the severity of GBS meningitis *in vivo*. After confirming that the BspC phenotypes observed within the FRT were also dependent on the same V-domain ligand-binding pocket, we hypothesized that carfilzomib treatment would limit GBS vaginal colonization *in vivo*. We first tested the ability of carfilzomib to block GBS aggregation. Similar to the results observed with V-domain protein blocking of GBS aggregation, carfilzomib treatment prevented aggregation of both WT and the complemented strain but did not impact the Δ*bspC* mutant (Fig. 7A). Next, we examined co-colonization of the murine vaginal lumen with GBS WT and the Δ*bspC* mutant, followed by daily treatment with either carfilzomib or the vehicle control directly into the vaginal lumen to ensure that any observed phenotype was BspC dependent. The carfilzomib treatment led to significantly faster clearance of WT GBS compared to the vehicle-treated controls after only 2 days of treatment (Fig. 7B) but did not impact the clearance of the Δ*bspC* mutant (Fig. 7C). We also investigated the impact of a carfilzomib treatment on GBS ascension to other tissues during WT colonization by inoculating the vaginal lumen with just WT GBS followed by treating with either carfilzomib or the vehicle control and harvesting tissues 48 h postinfection. Carfilzomib treatment led to decreased WT GBS in the vaginal lumen (Fig. 7D) and resulted in less GBS recovered from vaginal, cervical, and uterine tissues (Fig. 7E to G). Collectively, these data indicate that a carfilzomib treatment could be used to reduce GBS vaginal colonization and ascension in the FRT.

## DISCUSSION

The GBS surface proteins and host cell receptors that allow GBS to colonize the female reproductive tract and promote ascending infection are not well studied. Here, we show that the AgI/II protein, BspC, contributes to GBS adherence to human vaginal and cervical cells, as well as auto-aggregation on the surface of host cells. These phenotypes led to an increased ability to colonize the murine vaginal lumen and cause ascending infection resulting in increased neutrophil signaling within the vagina, cervix, and uterus. We also identify K19 as a host cell receptor for BspC, which is increased in surface localization on vaginal epithelial cells during GBS infection. Intermediate filament proteins such as K19 are typically thought of as predominantly cytoplasmic proteins that contribute mainly to the structural integrity of all cell types ([Bibr B37][Bibr B38][Bibr B39]). A large body of literature has emerged detailing the additional role of intermediate filament proteins in signal transduction within cells, where they interact either directly with the extracellular environment or through intermediaries to impact functions ranging from cell migration to altered immune response (31). K19 is known to be expressed by vaginal, ectocervical, and endocervical cells ([Bibr B40][Bibr B41][Bibr B42]); however, to our knowledge this is the first report confirming surface localization of K19 in the FRT during bacterial interaction. Interestingly, we observed additional ~32 kDa spots on the far Western blot that could be investigated further as an additional BspC receptor.

We recently demonstrated the role of BspC in the pathogenesis of GBS meningitis through its interaction with another intermediate filament protein, vimentin (15, 16). We observed a similar phenomenon where GBS infection altered vimentin organization in BBB endothelial cells, leading to increased vimentin at the cell surface allowing for interaction with BspC (16). We also demonstrated that endothelial cells used vimentin to alter chemokine expression and NOD2 activation in response to GBS infection (18). Our observations that GBS infection slightly increased overall protein levels of its own epithelial receptor, K19, and dramatically altered K19 localization toward the cell surface have many possible downstream effects on the host cell. K19 has been shown to modulate expression of ALDH1, CXCR4, CD133, and CXCL12, and impacts phosphorylation of Src and GSK3β (43, 44). K19 has also been implicated in cell cycle progression through the transcription factor E2F1 (45). It is currently unclear whether these additional cellular roles for K19 are impacted by interaction with GBS or BspC. Future studies should also aim to determine which GBS factors impact K19 localization.

Based on the previously determined interaction between BspC and vimentin (estimated dissociation constant [*K*_d_] of 3.39 μM [16]) compared to the interaction of BspC with K19 (estimated *K*_d_ of 32.9 nM), we speculate that K19 is the primary receptor for BspC used to promote GBS colonization in its commensal niche. The similarity between K19 and vimentin as closely related intermediate filament proteins may allow for incidental interaction of GBS with vimentin at other sites. We had previously shown that the V9 vimentin monoclonal antibody could block interaction with BspC (16). There is a 13-amino-acid stretch from E396 to E408 of vimentin that is immediately upstream of the known V9 antibody binding site (46) that has an 85% similarity to the E376 to E388 region of K19. This is the only conserved region between both proteins that is also close to the V9 vimentin antibody binding site, and therefore represents a likely interaction site for BspC. Interestingly, vimentin can be expressed in the FRT during the epithelial-to-mesenchymal transition (EMT). GBS infection has been shown to induce EMT within the vaginal tract, resulting in increased ascending infection (47). It is already known that vimentin is upregulated during EMT, which was also recently confirmed specifically during GBS-induced EMT at placental chorioamniotic membranes (48). Collectively, this suggests that the BspC–K19 interaction may allow GBS to establish colonization of the FRT, but during EMT induction, interaction with vimentin may allow GBS adherence to exfoliating cells, resulting in the observed increase in ascending infection. The increased inflammation caused by BspC also has potential to contribute to adverse pregnancy outcomes such as chorioamnionitis in this context as well, although this complex interplay warrants further investigation.

While we confirmed direct interaction between the BspC V-domain and K19 (estimated *K*_d_ of 362 nM), this interaction was ~10-fold weaker than what we observed with full-length BspC (estimated *K*_d_ of 32.9 nM). The V-domain of the S. mutans AgI/II SpaP required inclusion of parts of the adjacent A and P domains for optimal conformation (49), so the BspC V-domain may similarly require other domains for efficient binding to K19. However, previously published data demonstrated that the V-domain displayed expected folding using circular dichroism and interacted with vimentin with a comparable *K*_d_ to the full-length protein. Another possibility is that other portions of BspC may contribute to interaction with K19. It is known that domains other than the V-domain of AgI/II family proteins contribute to binding of various receptors (13, 14). Still, BspC mutants with nonfunctional V-domain pockets displayed severe defects in adherence and aggregation, implying that the V-domain pocket contributes significantly to these phenotypes.

Blocking the BspC V-domain with carfilzomib treatment promoted GBS clearance from the vaginal lumen (2.5 log reduction) and reproductive tract tissues (1.5 log reduction). Carfilzomib is an FDA-approved drug that targets the proteasome and is typically used in anticancer therapy. To consider the use of carfilzomib to reduce GBS persistence in the FRT, other parameters such as delivery method, dose, and side effects must also be considered. Carfilzomib itself is known to cause embryo-fetal toxicity in rabbits, which would likely preclude its use for treatment of pregnant women (50). Carfilzomib treatment has been shown to be well tolerated in human children as young as 1 year old (51), but to our knowledge, studies haven’t investigated the impact of carfilzomib on a fetus in the weeks immediately prior to birth. Further investigation of analogous drugs designed based on the R-groups involved with carfilzomib binding to the BspC V-domain but with less toxicity would be necessary before pursuing anti-BspC treatment.

In addition to blocking GBS interaction with K19, anti-BspC therapies may also impact polymicrobial interactions. Candida albicans is commonly found within the vaginal tract, and its presence is known to be a risk factor for GBS colonization ([Bibr B52][Bibr B53][Bibr B54]). Synergy between GBS and C. albicans in co-inoculation experiments has been shown to promote adherence of each to vaginal epithelial cells in a BspC-dependent manner (19, 20). The mechanism for this interaction is unknown; however, if the BspC V-domain is coordinating the interaction with C. albicans, then treatments like carfilzomib might help disrupt such polymicrobial communities and promote clearance of both opportunistic pathogens. These polymicrobial communities often grow in biofilm-like states, and AgI/II proteins are known to form functional amyloids that can provide a matrix to stabilize these communities ([Bibr B32][Bibr B33][Bibr B36]). Using anti-BspC strategies to disrupt these bacterial communities might also allow other therapeutics to more effectively clear GBS colonization prior to exposing the fetus and newborn.

**FIG 8:**
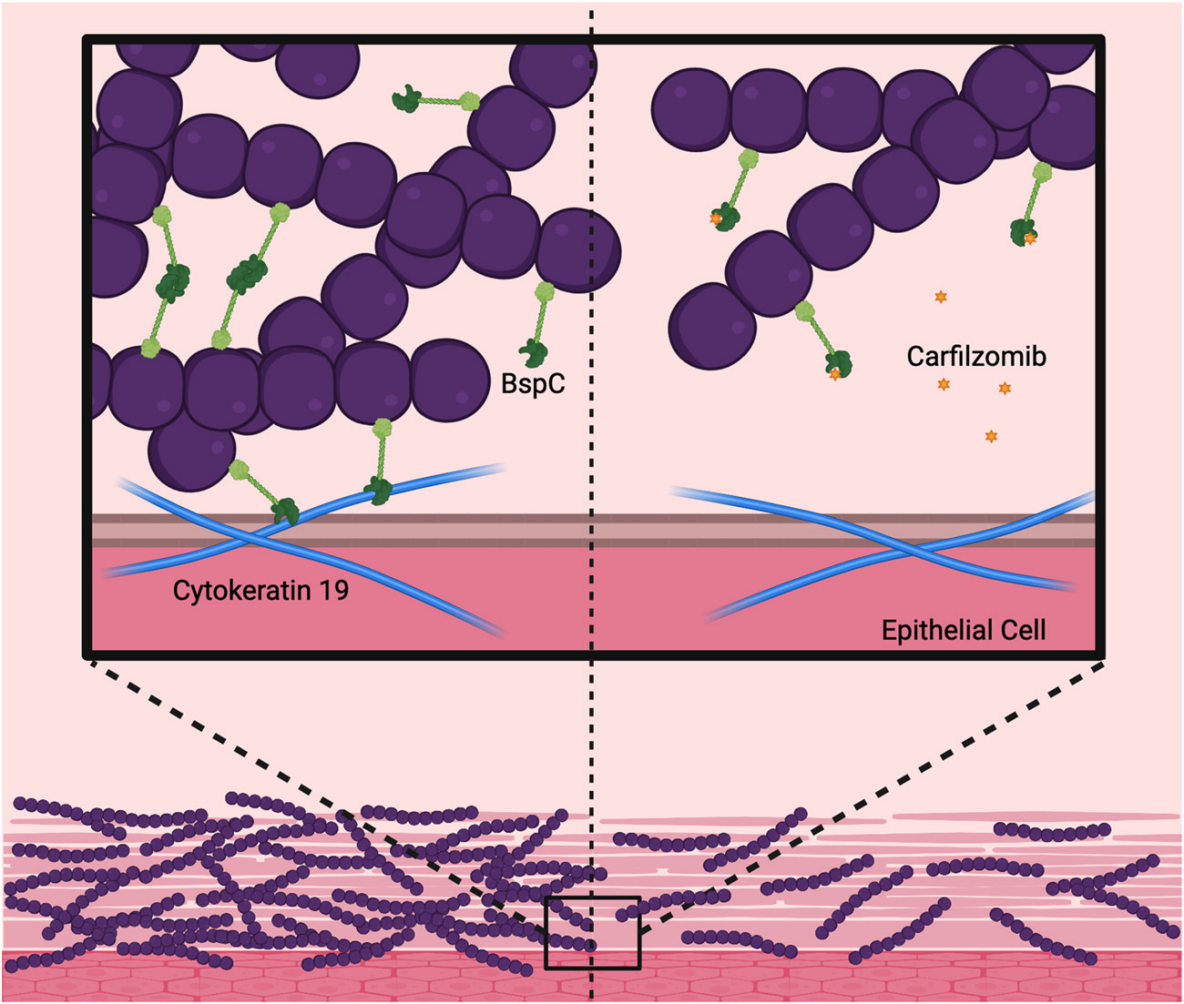
Role of BspC in the FRT. The BspC V-domain promotes GBS aggregation and interaction with host cytokeratin 19, contributing to adherence to the vaginal epithelium and colonization of the FRT. Therapeutic treatment with a drug such as carfilzomib that targets the BspC V-domain blocks its function and promotes GBS clearance from the FRT. Made using BioRender.

Our data reveal that BspC is an important adhesin used by GBS to assist in colonization of the FRT through interaction with K19 (Fig. 8). We have previously identified vimentin as a brain endothelial receptor for BspC (15, 16) and shown that GBS Srr-1 (9) mediated attachment to vaginal epithelial cells can be blocked with an antibody to cytokeratin 4 (K4). Collectively, this emerging body of evidence reveals that intermediate filament proteins may be an understudied yet conserved mechanism by which GBS exploits host proteins to promote colonization and disease. Importantly, this work also lays the foundation for development of therapeutics that block these interactions to promote GBS clearance and prevent disease.

## MATERIALS AND METHODS

### Ethics statement.

Animal experiments were approved by the committee on the use and care of animals at the University of Colorado School of Medicine (protocol #00316) and performed using accepted veterinary standards. The University of Colorado School of Medicine is AAALAC accredited, and the facilities meet and adhere to the standards in the *Guide for the Care and Use of Laboratory Animals* (55).

### Bacterial strains and growth conditions.

GBS clinical isolate COH1 (serotype III, ST-17) (21) and its isogenic Δ*bspC* mutant were used for most experiments. GBS strain 515 and the isogenic Δ*bspC* mutant were also used. For competition experiments, a rifampicin-resistant Δ*bspC* mutant strain was generated by spontaneous mutation after plating 10× concentrated log-phase GBS onto Todd Hewitt agar (THA) plates supplemented with rifampicin at 15 μg/mL. The resistant strain was then confirmed to have no significant difference to the parent Δ*bspC* strain in adherence to VK2 cells or growth (Fig. S1D and E). Antibiotics were added to media for selection of GBS strains containing pDCErm or pDESTErm at 5 μg/mL erythromycin. GBS strains were grown in Todd Hewitt broth (THB) static at 37°C.

### Microscale thermophoresis.

Full-length BspC and V-domain proteins were purified as previously described (15, 16). Briefly, full-length BspC was cloned into the pOPINF expression vector and the V-domain was cloned into the pTEV5 expression vector, both incorporating a His6 tag at the N terminus of the cloned product. Both proteins were expressed in E. coli BL21 (DE3) cells by growth in LB broth supplemented with 1 mM IPTG. For the full-length protein, cell pellets were lysed by sonication, centrifuged to remove debris, and purified by nickel affinity chromatography using a HiTrap IMAC column and size exclusion chromatography (SEC) using a Superdex 200 column (both from GE Healthcare). For the V-domain protein, cell pellets were lysed using the BugBuster Protein Extraction Reagent, centrifuged to remove debris, and purified by nickel affinity chromatography using a HIS-Select Nickel Affinity Gel Column (both by Sigma). The His6 tag was cleaved using the TEV protease and repurified using the HIS-Select Nickel Affinity Gel Column.

His6-Tagged cytokeratin-19 (Novus Biologicals) was labeled with RED-tris-NTA 2nd Generation dye (NanoTemper Technologies) according to the manufacturer’s instructions. Full-length or V-domain proteins were serially titrated and mixed at a 1:1 volume/volume ratio with labeled cytokeratin-19 from a concentration of 5 μM to 0.153 nM, while cytokeratin-19 was kept at a concentration of 25 nM. Measurements were performed in premium capillaries with a Monolith NT.115 Pico system at 20% excitation power.

### Cell culturing conditions and assays.

The well-characterized immortalized human cell lines representing vaginal epithelial cells (VK2/E6E7), ectocervical cells (ECT1/E6E7), and endocervical cells (END1/E6E7) (56) were obtained from the American Type Culture Collection (ATCC CRL-2616, ATCC CRL-2614, and ATCC CRL-2615, respectively) and were maintained in keratinocyte serum-free media (KSFM; Gibco) supplemented with 0.5 ng/mL human recombinant epidermal growth factor and 0.05 mg/mL bovine pituitary extract. Cells were grown at 37°C with 5% CO_2_.

Assays to determine the total number of cell surface-adherent bacteria were performed as describe previously (57). Briefly, bacteria were grown to mid-log phase and used to infect cell monolayers (1 × 10^5^ CFU, at a multiplicity of infection [MOI] of 1). Following 30 min of incubation, cells were washed 5× with phosphate-buffered saline (PBS) to remove nonadherent bacteria. Cells were detached with 0.25% trypsin-EDTA solution and then lysed with 0.025% Triton X-100 by vigorous pipetting. The lysates were then serially diluted and plated on THB agar to enumerate bacterial CFU. For protein-blocking assays, a VK2 monolayer was pretreated with V-domain protein at a concentration of 10 μM/well, or with the PBS vehicle control, and incubated for 30 min prior to infection with GBS. After infection, assays were continued as normal for enumeration of adherent bacterial CFU.

Aggregation on the surface of VK2 cells was observed by infecting a VK2 monolayer grown on a chamber slide with wild-type or Δ*bspC* strains expressing green fluorescent protein (GFP) (pDESTErm::GFP) as described above, and then incubating at 37°C with 5% CO_2_ for 24 h. After incubation, cells were washed 5× with PBS before the chambers were removed from the slide, and Fluoroshield Mounting Media containing DAPI was used to mount a coverslip. Slides were then imaged at ×20 magnification with a BZ-X710 fluorescence microscope (Keyence).

Infection assays to measure host transcripts were done by growing bacteria to midlog phase and then infecting cell monolayers (1 × 10^6^ CFU, at an MOI of 10). After 4 h of incubation at 37°C with 5% CO_2_, total RNA was extracted (Macherey-Nagel) and converted to cDNA (Quanta Biosciences) according to the manufacturers’ instructions. Primers for IL-8, CXCL-1, and GAPDH were utilized as previously described (58).

### K19 expression and localization cell assays.

K19 in VK2 cells was observed by infecting a VK2 monolayer grown on a chamber slide for microscopy or in a 6-well plate for flow cytometry and Western blots with wild-type COH1 as described above, and then incubating at 37°C with 5% CO_2_ for 24 h. For microscopy experiments, after incubation cells were washed 5× with PBS and blocked (10% green fluorescent protein [FBS] and 1% bovine serum albumin [BSA] in PBS) for 1 h before addition of the primary antibody (1:75 dilution of polyclonal rabbit anti-cytokeratin-19, Novus Biologicals) or IgG isotype control (Invitrogen). After 1 h at room temperature, cells were washed before addition of the secondary antibody (1:2,000 dilution of donkey anti-rabbit conjugated to Alexa Fluor 488, Invitrogen). After 45 min of incubation at room temperature, cells were washed and Fluoroshield mounting media containing DAPI was used to mount a coverslip. Slides were then imaged at ×20 magnification with a BZ-X710 fluorescence microscope (Keyence).

Flow cytometry experiments were done similarly as described above with the following changes. After infecting as described above, cells were washed with magnetic-activated cell sorting (MACS) buffer and collected using a cell scraper and then incubated with eBioscience Fixable Viability Dye eFluor 506 (Invitrogen) for 15 min prior to incubating with primary K19 antibody or IgG isotype control as described above, with the addition of a 1:400 dilution of eBioscience anti-human Fc receptor binding inhibitor polyclonal antibody (Invitrogen) for 1 h at room temperature. Cells were washed before addition of secondary antibody for 45 min at room temperature. Cells were washed and then fixed with paraformaldehyde (PFA) for 15 min. Cells were washed again and measured using a cytoFLEX flow cytometer. Analysis of K19 surface localization was done by gating on singlets that were negative for the viability dye and measuring AF488.

Cells for Western blot experiments were grown, infected, and collected in the same manner as the flow cytometry experiments. Cells were lysed by addition of Laemmli sample buffer (Bio-Rad) and incubation at 100°C for 5 min. Equal protein amounts were separated using an SDS-PAGE gel, which was transferred to a polyvinylidene difluoride (PVDF) membrane (Bio-Rad) and blocked with Intercept Blocking Buffer TBS (LI-COR) for 1 h at room temperature. Primary antibodies (K19 1:75 and GAPDH 1:1000, CST) were diluted in blocking buffer and incubated at room temperature for 1 h before washing. Secondary antibodies (IRDye 1:15,000, LI-COR) were added for 1 h at room temperature. Blots were developed using LI-COR Odyssey per the manufacturer’s instructions.

### Bacterial aggregation assays.

To quantify bacterial aggregation, overnight cultures of GBS were standardized to an OD600 of 1 in PBS and then vortexed vigorously for 10 s. Ten μL was taken from the top of each tube immediately, diluted 1:10 in PBS, and then the OD600 of each sample was read using a TECAN plate reader. This was repeated every 15 min without agitation of the tubes for a total of 90 min, and OD600 values were plotted as a percentage of the OD600 at time zero.

### Far Western blot and mass spectrometry.

Far-Western and mass spectrometry was done as previously described (16). Briefly, membrane proteins from VK2 cell lysates were enriched using a FOCUS membrane protein kit (G Biosciences), dissolved in rehydration buffer (7M urea, 2M thiourea, 1% tributylphosphine [TBP], and 0.2% ampholytes 3 to 10 NL), and quantified using 2D Quant kit (GE Healthcare). One hundred μg of proteins were loaded on 7-cm long immobilized pH gradient (IPG) strips with nonlinear gradient (NL) 3 to 10 pH gradient (GE Healthcare). Isoelectric focusing was done with the Multiphor II electrophoresis system (GE Healthcare) in three running phases (phase 1: 250V/0.01h; phase 2: 3500V/1.5h; and phase 3: 3500V/4.5h). The second-dimension SDS-PAGE was done using two 12.5% acrylamide gels. One was stained with Coomassie blue G250 (Bio-Rad, Hercules, CA) for mass spectrometry analysis. The other gel was transferred to a PVDF membrane for far-Western blot analysis.

The PVDF membrane was incubated in a blocking solution (5% skim milk in PBS) for 1 h. Recombinant BspC was biotinylated using an EZ-Link Sulfo-NHS-Biotin kit (ThermoFisher Scientific, Waltham, MA). The PVDF membrane was probed with the biotinylated BspC (100 μg) in a blocking solution overnight at 4°C. After washing, the PVDF membrane was incubated with an antibody conjugated to streptavidin-horseradish peroxidase (HRP). Interacting proteins were detected by adding enhanced chemiluminescence (ECL) reagents (ThermoFisher Scientific) and visualized by X-ray film exposure. The protein spots from far-Western blot were aligned to the corresponding protein spots in the Coomassie stained gel. The largest and darkest identified spot was excised and digested in gel with trypsin (Worthington). Peptide mass spectra were collected on MALDI-TOF/TOF (ABI 4700, AB Systems), and protein identification was performed using the automated result dependent analysis (RDA) of ABI GPS Explorer software V3.5. Spectra were analyzed by the Mascot search engine using the Swiss protein database.

### Mouse model of vaginal colonization and ascending infection.

We utilized our well-established murine model for GBS vaginal colonization ([Bibr B22][Bibr B23][Bibr B25]). The estrus cycles of 7- to 10-week-old female CD-1 or 129S mice obtained from Charles River Labs were synced via i.p. injections of 0.5 mg of β-estradiol in sesame oil. The following day, ~2 × 10^7^ CFU of GBS was inoculated directly into the vaginal lumen of the mice in 10 μL of PBS. Mice were then either swabbed with a sterile ultrafine swab or lavaged with PBS prior to plating on GBS CHROMagar plates for CFU enumeration. At the experimental endpoint, mice were euthanized, and their vaginal tract, cervix, and uterus were homogenized in PBS and plated on GBS CHROMagar for tissue CFU enumeration. Tissue homogenates were also used to measure KC protein concentration according to the R&D Systems ELISA kit as per manufacturer’s instructions.

For competition experiments, a 1:1 mix of wild-type COH1 and the rifampicin-resistant Δ*bspC* strain with a total of ~2 × 10^7^ CFU of GBS per 10 μL of PBS was used. CFU enumeration in the competition experiments was done by plating on GBS CHROMagar and THA supplemented with μg/mL rifampicin.

For carfilzomib treatment experiments, mice were inoculated with GBS as described above and then treated with 35 μg of carfilzomib (Fisher Scientific) or the vehicle (10% DMSO, 40% polyethylene glycol [PEG] 1500, 5% Tween 80, and 45% PBS) in 10 μL directly into the vaginal lumen. This treatment was given every 24 h until the experimental endpoint.
