# Supplementary figures and images for: The Group B Streptococcal Adhesin BspC Interacts with Host Cytokeratin 19 To Promote Colonization of the Female Reproductive Tract
Source: mBio. 2022 Sep 7;13(5):e01781-22. doi: 10.1128/mbio.01781-22 (PMC9600255; doi:10.1128/mbio.01781-22)

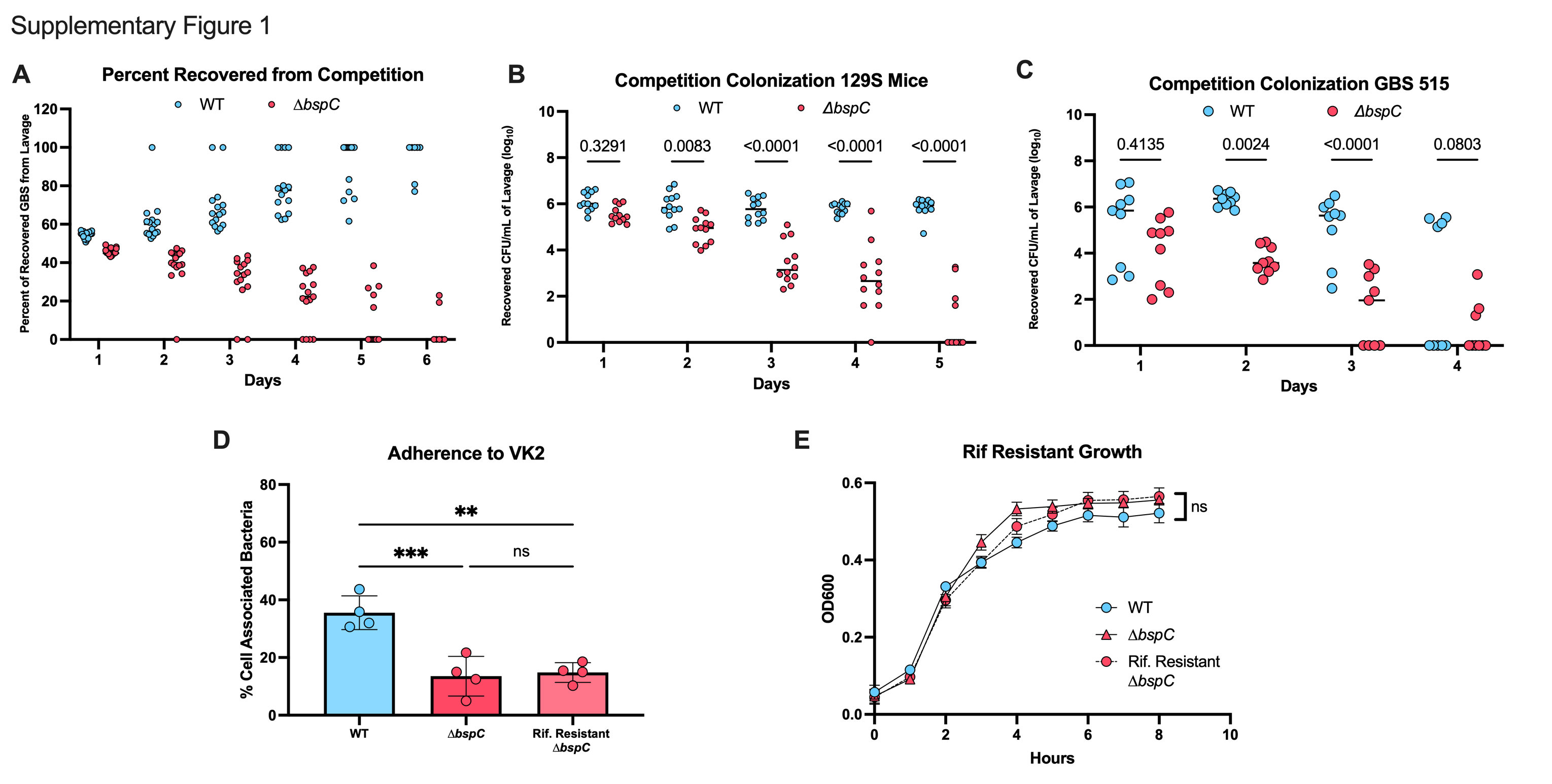

Supplement: FIG S1 [file mbio.01781-22-s0001.tif]

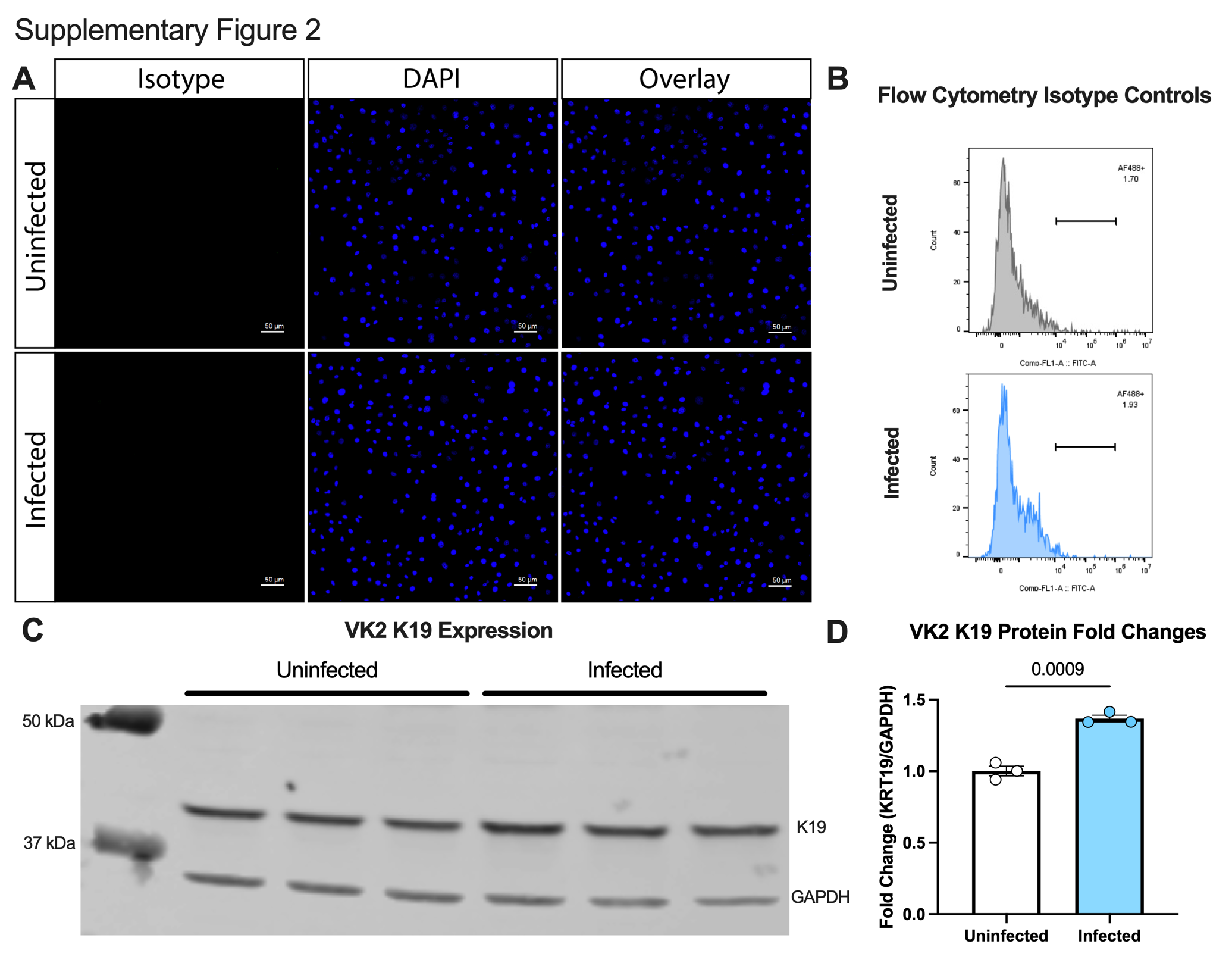

Supplement: FIG S2 [file mbio.01781-22-s0002.tif]
